# Co-occurrence network analysis unveils the actual differential impact on the olive root microbiota by two Verticillium wilt biocontrol rhizobacteria

**DOI:** 10.1186/s40793-023-00480-2

**Published:** 2023-03-22

**Authors:** Martina Cardoni, Antonio J. Fernández-González, Antonio Valverde-Corredor, Manuel Fernández-López, Jesús Mercado-Blanco

**Affiliations:** 1 Departamento de Protección de Cultivos, Instituto de Agricultura Sostenible, Consejo Superior de Investigaciones Científicas [CSIC], Córdoba, Spain; 2 Departamento de Microbiología del Suelo y la Planta, Estación Experimental del Zaidín, CSIC, Granada, Spain

**Keywords:** Biological control agents, Network topology, *Olea europaea*, *Paenibacillus polymyxa*, *Pseudomonas simiae*, Root microbial community, *Verticillium dahliae*

## Abstract

**Background:**

Verticillium wilt of olive (VWO), caused by *Verticillium dahliae* Kleb, is one of the most threatening diseases affecting olive cultivation. An integrated disease management strategy is recommended for the effective control of VWO. Within this framework, the use of biological control agents (BCAs) is a sustainable and environmentally friendly approach. No studies are available on the impact that the introduction of BCAs has on the resident microbiota of olive roots. *Pseudomonas simiae* PICF7 and *Paenibacillus polymyxa* PIC73 are two BCAs effective against VWO. We examined the effects of the introduction of these BCAs on the structure, composition and co-occurrence networks of the olive (cv. Picual) root-associated microbial communities. The consequences of the subsequent inoculation with *V. dahliae* on BCA-treated plants were also assessed.

**Results:**

Inoculation with any of the BCAs did not produce significant changes in the structure or the taxonomic composition of the ‘Picual’ root-associated microbiota. However, significant and distinctive alterations were observed in the topologies of the co-occurrence networks. The introduction of PIC73 provoked a diminution of positive interactions within the ‘Picual’ microbial community; instead, PICF7 inoculation increased the microbiota’s compartmentalization. Upon pathogen inoculation, the network of PIC73-treated plants decreased the number of interactions and showed a switch of keystone species, including taxa belonging to minor abundant phyla (*Chloroflexi* and *Planctomycetes*). Conversely, the inoculation of *V. dahliae* in PICF7-treated plants significantly increased the complexity of the network and the number of links among their modules, suggestive of a more stable network. No changes in their keystone taxa were detected.

**Conclusion:**

The absence of significant modifications on the structure and composition of the ‘Picual’ belowground microbiota due to the introduction of the tested BCAs underlines the low/null environmental impact of these rhizobacteria. These findings may have important practical consequences regarding future field applications of these BCAs. Furthermore, each BCA altered the interactions among the components of the olive belowground microbiota in idiosyncratic ways (i.e. PIC73 strongly modified the number of positive relations in the ‘Picual’ microbiota whereas PICF7 mostly affected the network stability). These modifications may provide clues on the biocontrol strategies used by these BCAs.

**Supplementary Information:**

The online version contains supplementary material available at 10.1186/s40793-023-00480-2.

## Background

The cultivated olive tree (*Olea europaea* L. subsp. *europea*) is a long-living woody plant of unquestionable economic, social and environmental significance. It is extensively grown in Mediterranean-type climate regions worldwide [1]. The intensification of olive cultivation observed in the last decades, mostly due to a growing demand for olive oil [2] may explain, among other reasons, the increase in both incidence and severity of olive pests and diseases [1, 3]. Among the biotic constraints affecting olive cultivation, the soil-borne fungus *Verticillium dahliae* Kleb., causal agent of Verticillium wilt of olive [VWO], is considered the most threatening disease in many areas where this tree is cultivated [4]. The effective control of VWO is very difficult due to a multiplicity of factors [5, 6], and available measures have so far proven unsuccessful when implemented individually. Therefore, an integrated disease management strategy is recommended [4]. The use of biological control agents [BCAs] to reduce the impact of VWO on susceptible cultivars or increase the durability of tolerant genotypes is a sustainable and environmentally friendly management approach in this context [7–11].

Our previous works showed the effectiveness of some olive rhizobacteria belonging to the genera *Pseudomonas* [12, 13] and *Paenibacillus* [7] to control VWO. One of the best performing BCA against VWO is *Pseudomonas simiae* PICF7 [14, 15], a versatile endophytic rhizobacteria isolated from olive roots (cultivar Picual) that displays biocontrol and plant-growth promoting (PGP) abilities in different plants [3, 7, 16–19]. This strain can colonize and endure within olive root tissues, triggering a broad range of defence responses in both roots [20] and above-ground tissues [3], although niche (i.e., roots/rhizosphere) competition seems to play a key role in the effective biocontrol of *V. dahliae* [21]. Another effective rhizobacteria controlling VWO is *Paenibacillus polymyxa* PIC73, originating from ‘Picual’ roots as well [7]. This strain also showed in vitro inhibition ability against a broad range of olive pathogens (i.e., *V. dahliae, Rosellinia necatrix, Phytophthora cinnamomi, Pseudomonas savastanoi* pv. *savastanoi*, *Colletotrichum nymphaeae* and *C. godetiae*) [7]. Several strains of *Paenibacillus* spp. are able to produce antimicrobials active against both bacteria and fungi, and a range of hydrolytic enzymes for the degradation of cellulose-containing cell wall components [22, 23].

Recent studies showed that inoculation with plant-associated beneficial bacteria may perturb indigenous microbial populations [24–26]. Even though the soil microbial community might have the ability to reorganize and return to the original state (resilience) after the disturbance provoked by the inoculation, the potential ecological impacts of microbial inoculants on the soil resident communities remain largely unknown. Indeed, the quick disappearance of a bacterial inoculum does not necessarily imply the lack of a lasting legacy over the soil indigenous community [25]. Biocontrol agents do not only affect the host (e.g., triggering genetic defence responses) and the pathogen (e.g., antibiosis), but they also have an effect on the plant-associated microbiome [27–31]. The impact of introducing a BCA on the natural pre-existing microbiota can take place at different levels, namely the structure and composition of the microbial communities, the functioning of their components, and/or the co-occurrence interaction networks. These outcomes have been recently highlighted in the case of the interaction of *P. simiae* PICF7 with the banana (Grand Naine Cavendish cultivar) root-associated microbiome [32]. Yet, our knowledge about the impact that BCA application has on the indigenous plant microbial communities is very limited, particularly in the case of the interactions taking place among their components. Understanding the microbe–microbe interactions is crucial in order to predict the consequences of these interactions for plant performance and physiology [33, 34]. The analysis of microbial co-occurrence interactions can also provide useful information about relevant members of the plant microbiota in order to improve plant health and counteract potential threats more effectively.

No studies are available on the impact that the introduction of BCAs has on the resident root microbial community of olive plants. Therefore, the main objectives of this work were: (I) to evaluate whether, and to what extent, the inoculation with two well-known BCAs, *P. simiae* PICF7 and *P. polymyxa* PIC73, modify the structure, the composition and the co-occurrence interactions of the belowground microbial communities associated to the olive cultivar Picual (VWO-susceptible); and (II) to assess the effect that the inoculation with *V. dahliae* has on the root-associated microbiota of ‘Picual’ plants previously bacterized with these BCAs. We tested the hypothesis that the introduction of PICF7 or PIC73 produces distinctive modifications in the composition and network topology of the ‘Picual’ root microbial community. The in-depth analysis of the changes produced in the resident microbiota may provide clues on the biocontrol strategies used by these BCAs.

## Methods

### Plant material and bacteria inoculation

Two hundred and fifty olive plants (cv. Picual, 2-year old) purchased in a commercial nursery (Córdoba, Spain) were grown in pots (11 × 11 × 12 cm), containing a peat-based substrate [35]. Prior to bacterial treatment, plants were acclimated in a greenhouse for one month under natural lighting and day/night temperature of 26 ± 2 °C, and relative humidity ranging from 40% (day) to 80% (night). *Pseudomonas simiae* PICF7 and *P. polymyxa* PIC73 inoculum were prepared as previously described [7, 36]. Bacterial inoculum consisted of 150 ml of a cell suspension (2.1 × 10^8^ cfu/mL in the case of PICF7 or 5.5 × 10^5^ cfu/mL for PIC73) that were added (by irrigation) to each pot accordingly to the experimental design (see below). Non-inoculated plants (control) were just drenched with 150 ml of sterile MgSO_4_·7H_2_O 10 mM. One week after bacterization, plants were inoculated with *V. dahliae* V937I, an isolate representative of the defoliating (D) pathotype [37], by adding 150 ml per pot of a conidia suspension (1 × 10^6^ conidia/mL) prepared as previously described [21]. Plants of the control treatment (MgSO_4_·7H_2_O) were drenched with 150 ml of water in this step.

### Experimental design

**Fig. 1:**
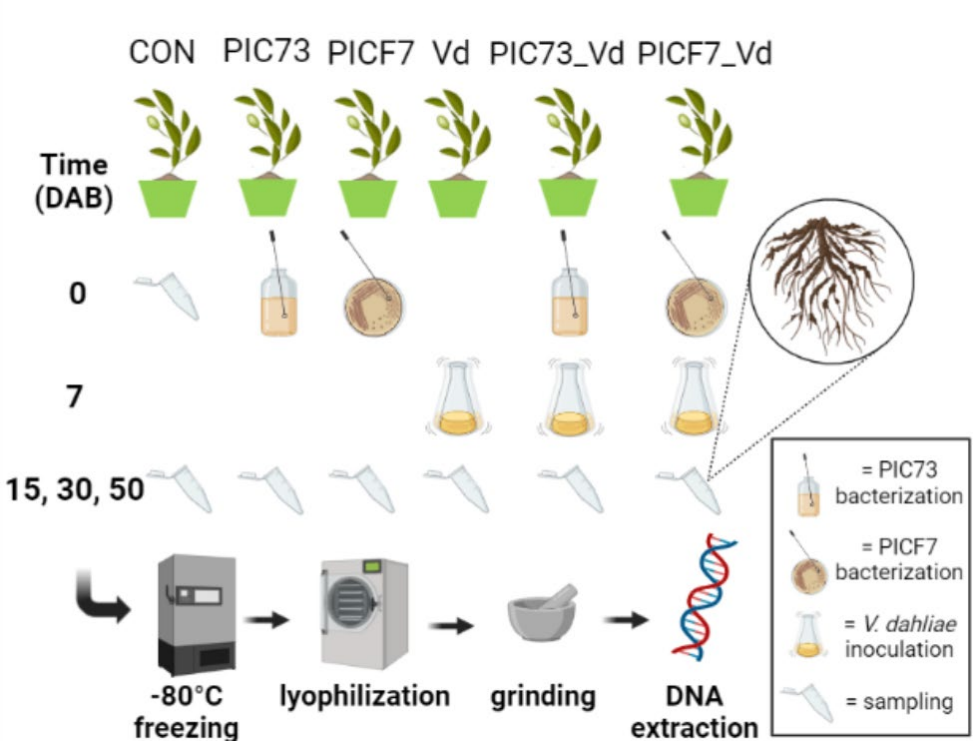
Experimental design of the olive-BCAs-*Verticillium dahliae* bioassay and the sampling strategy followed

The bioassay consisted of the following treatments: (1) 40 control, non-inoculated plants (CON); (2) 30 plants bacterized with *P. polymyxa* PIC73 (PIC73); (3) 30 plants treated with *P. simiae* PICF7 (PICF7); (4) 30 plants inoculated with *V. dahliae* V937I (Vd); (5) 30 plants bacterized with PIC73 and subsequently inoculated with V937I (PIC73_Vd); and (6) 30 plants treated with PICF7 and then inoculated with V937I (PICF7_Vd) (Fig. 1). Additionally, 10 plants for each treatment were used to monitor the presence of VWO symptoms during the experiment. The study was performed at three different levels separately: (i) “Treatment” (i.e., bacterized, inoculated with *V. dahliae*, bacterized + inoculated with *V. dahliae* and non-inoculated/non-bacterized), (ii) “Time” (t0 and 15, 30 and 50 days after bacterization, DAB), and (iii) “Treatment-Time” (all treatments at each single sampling time). Root tissues were sampled at 0 (only for control plants), and at 15, 30 and 50 (ten plants per time-point and treatment) DAB. Root samples were collected and washed gently under tap water to remove the excess substrate without losing particles firmly attached to the root. The roots were then stored at -80 degrees Celsius until further processing (Fig. 1).

At the first sampling time (i.e., at 0 days after bacterization, DAB) 10 non-inoculated (control) plants were sampled and 120 plants were treated with microorganisms as follows: (i) 60 plants were bacterized with *Paenibacillus polymyxa* PIC73 (i.e., plants of the PIC73 and PIC73_Vd treatments), and (ii) 60 plants were bacterized with *Pseudomonas simiae* PICF7 (i.e., plants of the PICF7 and PICF7_Vd treatments). At the second sampling time (i.e., 7 DAB) 90 plants were inoculated with *Verticillium dahliae* V937I (i.e., plants of the Vd, PIC73_Vd and PICF7_Vd treatments, 30 plants per treatment). At the remaining sampling times (i.e., at 15, 30 and 50 DAB) ten plants of each treatment were sampled. Samples consisted of the entire root system and the potting substrate firmly attached to it. At each sampling time, collected samples were immediately frozen and stored at -80° C. At the end of the experiment all samples were lyophilized, ground to a fine powder and subjected to the DNA extraction procedure.

### DNA extraction and illumina sequencing

Lyophilized root samples were ground to a fine powder using sterile mortars and pestles. DNA from 100 mg of ground root tissues per sample was extracted using the Maxwell RSC (Rapid Sample Concentrator) with the ‘PureFood GMO and Authentication’ Kit (Promega Corporation, Madison, WI, USA), according to the manufacturer’s instructions. DNA quality was then checked as previously described [32]. The DNA from root tissues was sequenced using the 2 × 300 PE technology in Illumina MiSeq platform at the genomics service of the Institute of Parasitology and Biomedicine “López Neyra” (CSIC), Granada, Spain. In the first run, prokaryotic libraries were constructed by amplifying the hyper-variable V3-V4 regions of the *16 S rRNA* gene. In the second run, eukaryotic libraries were constructed by amplifying the ITS2 region. Detailed information about the sequencing strategy has been previously reported [34].

### Illumina data processing

Raw reads were processed using DADA2 [38]. The Micro4all tutorial for *16 S rRNA* and ITS2 amplicons processing was followed [39]. Only two samples that did not reach the 10.000 reads (i.e., one belonging to the group of plants bacterized only with PICF7 and another bacterized with PICF7 and then inoculated with *V. dahliae*, both at 15 DAB) had to be eliminated from the analyses. The classification of bacterial and fungal amplicon sequence variants (ASVs) was achieved using the *assignTaxonomy* command against the Ribosomal Database Project II, training set v.18 [40] and the UNITE v.7.2 dynamic database, respectively [41]. All ASVs classified as mitochondria, chloroplast and unknown sequences were removed. ASVs accounting for less than 0.0168 and 0.005% of the total sequences were removed for bacteria and fungi, respectively. These percentages were calculated according to the MOCK community used (ZymoBIOMICS Microbial Community Standard II (Log Distribution), ZYMO RESEARCH, CA, United States) and to the recommendations by Bokulich et al., [42].

### Statistical analysis

All analyses were performed following the above-mentioned tutorial [39]. Alpha diversity indices [observed richness, Shannon, inverse of Simpson diversity (InvSimpson) and Evenness (Pielou index) were compared in rarefied samples with one-way Analysis of Unbalanced Variance using the R package *car* [43] and with the Kruskal–Wallis test. The Tukey honestly-significant-difference and Wilcoxon signed rank were used as *post hoc* tests. For the beta diversity, the normalized data, obtained using the Bio-Conductor package *edgeR* [44], were considered to perform the permutational analysis of variance (PERMANOVA) using three different distance methods: Bray-Curtis, Unifrac and Weighted-Unifrac. A permutational analysis of multivariate homogeneity of groups dispersions (BETADISPER) was also performed [34]. Pairwise differences between groups were assessed with the function *pairwise.adonis* (R package *pairwiseAdonis*). The PERMANOVA significant results were plotted by Non-metric MultiDimensional Scaling analysis (NMDS) and Principal Coordinates Analysis (PCoA) using all the three distance methods mentioned above. Biologically relevant microbial phyla and genera were obtained, according with their statistically significant differences in relative abundance, using the R package *ANCOMBC* [45].

### Network construction

Microbial (bacterial and fungal) networks were separately constructed for each treatment (control, PICF7-bacterized, PIC73-bacterized, *V. dahliae*-inoculated, PICF7/*V. dahliae*-inoculated and PIC73/*V. dahliae*-inoculated) considering all sampling times together (n = 40 for the control, n = 30 for PIC73-bacterized and PIC73/*V. dahliae*, n = 29 for PICF7-bacterized and PICF7/*V. dahliae*-inoculated). The networks were built and drawn by using the MENAP website and Cytoscape v.3.9.1 as previously described [34].

## Results

### Characteristics of sequencing datasets

A total of 9,936,065 (bacterial) and 5,566,966 (fungal) good quality reads were retained after the clustering. To avoid an overestimation of the diversity, ASVs with less than 0.005% of the high-quality reads were discarded. Therefore, a total of 693 bacterial and 511 fungal ASVs were considered.

### Alpha and beta diversities of the ‘Picual’ root microbiota are not significantly affected by the presence of the BCAs or the inoculation with ***Verticillium dahliae***

Concerning alpha diversity, bacterial and fungal communities showed similar trends for all the indices considered. No significant difference was found considering the factor “Treatment”, and both communities showed a significant increase over time for the number of ASVs (Observed richness) and Shannon index (*p* < 0.001) (Table 1). Interesting to note the significant difference (*p* < 0.05 Dunn *post-hoc* test) observed between the first (t0 for the control and 15 DAB for the other treatments) and the last sample time (50 DAB) for all treatments, except for PIC73 (Figures S1 and S2, Additional file 1).

Regarding to beta diversity the bacterial and fungal communities showed different trends during the experiment (Table 2). For bacteria, despite the fact that the PERMANOVA test showed statistically significant differences for all the factors considered, pairwise comparisons and the PCoA analysis performed on Bray–Curtis distances (Figures S3, S4, S5, Additional file 1) did not confirm such differences. Concerning the fungal community, and in spite of statistically significant PERMANOVA results for the factor “Treatment” (*p* < 0.05) (Table 2), pairwise comparisons did not show relevant differences. In contrast, the PCoA analysis confirmed the results of PERMANOVA that showed the influence of time on the mycobiome (Figure S6, Additional file 1). Indeed, the fungal community displayed differences between 0 and 50 DAB regardless the treatment.

**Table 1:** *p* values from Kruskal-Wallis tests of alpha richness and diversity indices considered in this study

Bacteria					Fungi			
Analysis level	Observed richness	Shannon	Inv. Simpson	Evenness	Observed richness	Shannon	Inv. Simpson	Evenness
Treatment	0.29	0.96	0.89	1	**< 0.05**	0.57	0.75	0.98
Time	**< 0.001**	**< 0.001**	**< 0.001**	**< 0.05**	**< 0.001**	**< 0.001**	0.08	0.19
Treatment-Time	**< 0.001**	**< 0.05**	0.10	0.42	**< 0.001**	**< 0.05**	0.63	0.83

**Significant** ***p*** **values are shown in boldface**

**Table 2:** PERMANOVAs of quantitative beta diversity index

	Bacteria			Fungi		
Analysis level	Bray-Curtis		BETADISPER	Bray-Curtis		BETADISPER
	R2	p-value	p-value	R2	p-value	p-value
Treatment	0.05	< 0.001	0.79	0.03	< 0.05	0.08
Time	0.06	< 0.001	**< 0.01**	0.08	**< 0.001**	**< 0.05**
Treatment-Time	0.17	< 0.001	0.34	0.16	**< 0.001**	**< 0.05**

**Significant** ***p*** **values from pairwise adonis tests are shown in boldface**

### The composition of the ‘Picual’ root microbiota only shows minor significant alterations upon bacterization with the BCAs and inoculation with ***V. dahliae***

**Fig. 2:**
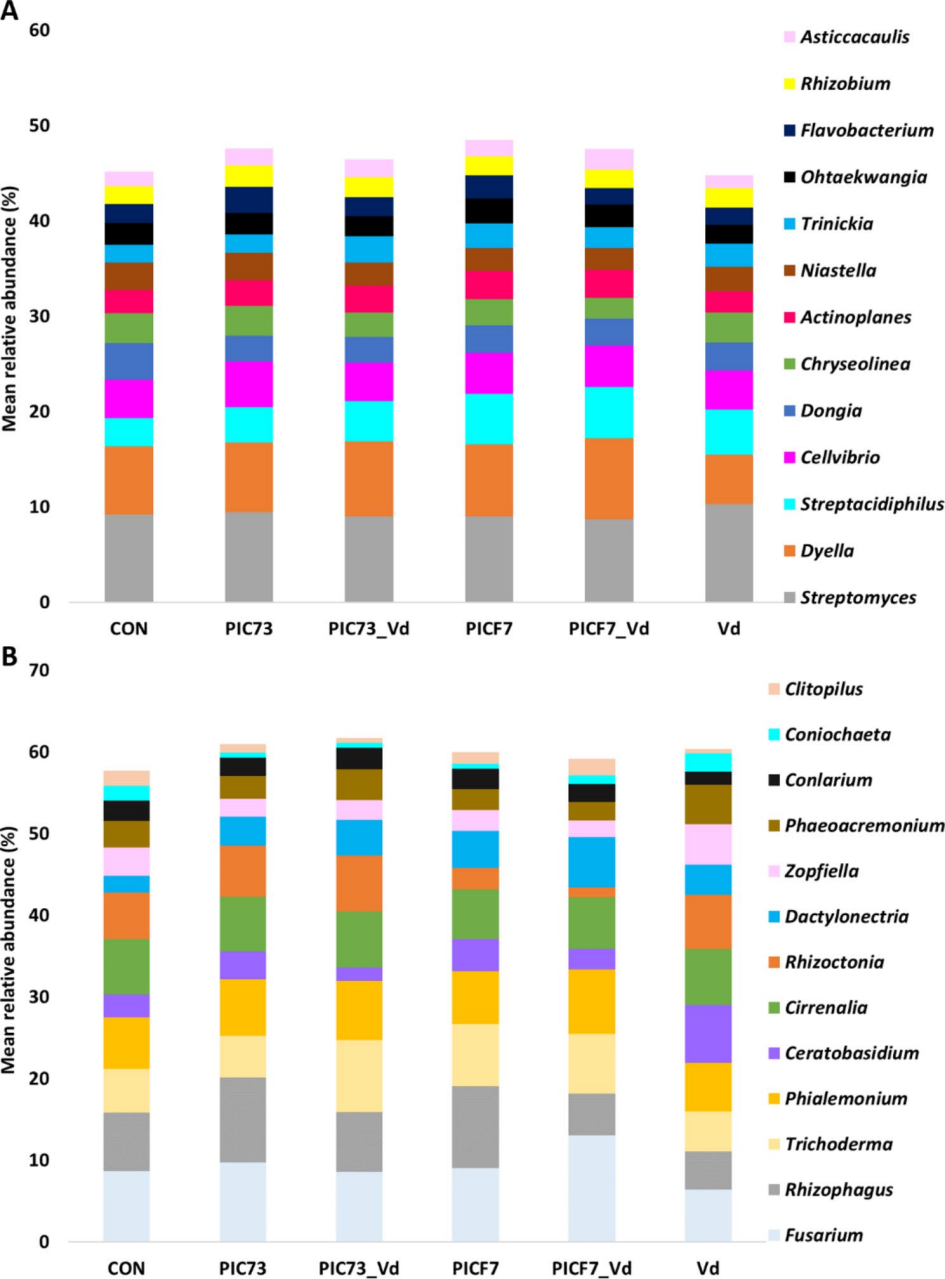
Taxonomy profiles at the genus level of the ‘Picual’ root-associated microbiota

The bacterial taxonomic profile was dominated by *Proteobacteria*, representing no less than 55% of the relative abundance, followed by *Actinobacteria* (21%) and *Bacteroidetes* (15%). Few significant differences were found among treatments considering both all sampling times altogether and separately. At the “Treatment” level, only a significant decrease of *Verrucomicrobia* and *Acidobacteria* was observed in PICF7 compared with the control (Fig. S7A, Additional file 1). Considering the treatments at each sampling time most of the significant differences were detected for minor phyla (relative abundance < 0.1%) (Figure S8A, Additional file 1; Additional file 2). At genus level, most of the significant changes observed also affected minor taxa (relative abundance < 1%) (Additional file 3). Nevertheless, some major taxa (relative abundances > 1%) showed differences that are worth mentioning. For instance, considering only the treatments, increases of *Streptacidiphilus* in all PICF7-treated samples (i.e., in the presence and absence of the pathogen) (ANCOMB *p* = 0.049 and 0.008 for PICF7 and PICF7_Vd, respectively) and of *Rhizobium* in the PIC73 treatment were detected (ANCOMB *p* = 0.019) (Fig. 2A). Furthermore, decrease of *Dyella* was observed in *V. dahliae*-inoculated plants compared with all treatments (Fig. 2A) (ANCOMB *p* < 0.05). Taking into account also the time, the most noticeable change for major taxa was observed at 15 DAB (Additional file 4). At this sampling time, plants treated with PICF7 presented a significant increase of *Streptacidiphilus* compared with the control (ANCOMB *p* < 4.79•10^− 6^), and plants inoculated with PIC73 showed increments in the relative abundance of *Rhizobium* and *Flavobacterium* (ANCOMB *p* = 7.11•10^− 4^ and 2.48•10^− 7^, respectively) (Figure S9, Additional file 1). Interestingly, plants bacterized with PICF7 and then inoculated with *V. dahliae* showed a significant increase of *Novosphingobium* at 15 DAB compared with all the other treatments (ANCOMB *p* < 0.05) (Figure S9, Additional file 1).

With regard to the fungal dataset the most abundant phyla were *Ascomycota*, *Basidiomycota* and *Glomeromycota*, accounting for at least 97% of the total sequences. The comparison among treatments revealed that plants inoculated with *V. dahliae* showed a decrease in *Glomeromycota*, although this difference was only statistically significant versus the PIC73 treatment (ANCOMB *p* = 0.003) (Figure S7B, Additional file 1). Considering each sampling time, significant changes were mostly found in minor phyla (relative abundance < 0.1%) (Figure S8B, Additional file 1; Additional file 5). The same trend was observed at the genus level (Fig. 2B and Figure S10, Additional file 1) (Additional file 6), except for the major genera (relative abundance > 1%) *Rhizoctonia* considering all the sampling times. It was present in the PICF7 treated plants at 15 and 30 DAB and absent at 50 DAB, while it showed the reverse tendency in PICF7_Vd samples (ANCOMB *p* < 0.05) (Figure S10, Additional file 1).

Bacterial (A) and fungal (B) taxonomic profiles at genus level for the different treatments considered in this study: control (CON], *Paenibacillus polymyxa* PIC73-treated (PIC73), *P. polymyxa* PIC73/*V. dahliae*-inoculated (PIC73_Vd), *Pseudomonas simiae* PICF7-treated (PICF7), *P. simiae* PICF7/*V. dahliae*-inoculated (PICF7_Vd), and *V. dahliae*-inoculated (Vd). Only the genera with a relative abundance > 2% are shown (n = 10). Asterisks indicate the genera that showed significant differences (ANCOMB *p* < 0.05) by the taxonomical analysis (see main text).

### The biocontrol strains PIC73 and PICF7 produced distinctive alterations in the topology of the ‘Picual’ root microbiota co-occurrence network

**Fig. 3:**
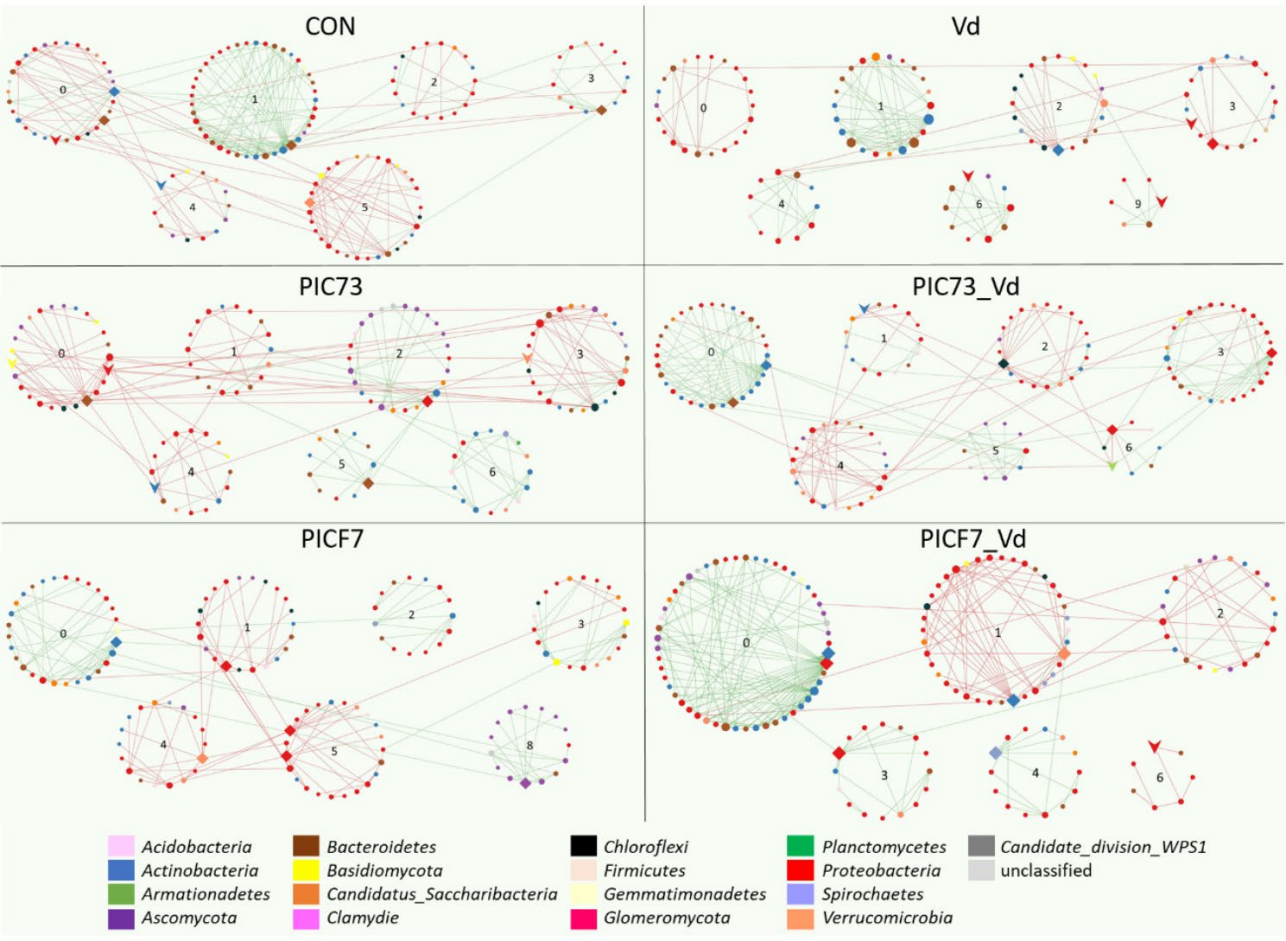
Co-occurrence networks of the ‘Picual’ root-associated microbiota of each treatment considered in this study

The introduction of the BCAs provoked a reduction of the network complexity and the increase of the distance among nodes compared to the control treatment. This shift in the network topology was more evident in the presence of PIC73. Indeed, smaller number of total links, lower average degree of nodes (avgK) and higher geodesic distance (GD) values were observed (Table 3). The network of the PIC73 treatment showed the lowest average clustering coefficient (avgCC) and percentage of positive edges (PEP) values denoting decreasing of connectivity and increasing in the number of negative interactions compared with the control (Table 3; Fig. 3). These changes were not observed after PICF7 inoculation, whose network otherwise showed the highest modularity (Table 3). Interestingly, the keystones detected in the PIC73 network were not observed in the other networks, while the PICF7 network presented one module hub identical to that of the control network (*Verrucomicrobia*/*Aterococcus*), and absence of connectors (Table 4). The networks of plants bacterized with BCAs showed fungi among their keystone. Thus, the genus *Clitopilus* (*Basidiomycota*) was a connector in the PIC73 network, while an unidentified *Ascomycota* genus acted as module hub in the PICF7 network (Table 4).

### ***Verticillium dahliae*** reduces the complexity of the ‘Picual’ root microbiota co-occurrence network

Similarly to the introduction of the BCAs, the network of the *V. dahliae*-inoculated plants showed less complexity compared with the control treatment. Moreover, this network presented the lowest number of total nodes and links among all networks analysed. It also showed higher modularity, more distance among modules [i.e., less interaction/edges among them], and major compartmentalization compared with the control one (Table 3). Finally, a huge abundance of *Proteobacteria* representatives were observed as keystone species for the Vd network (Table 4).

### Co-occurrence networks respond differently to the ***V. dahliae*** inoculation depending on the previously-introduced BCA

The inoculation of *V. dahliae* in PIC73-treated plants produced an increase in modularity (Table 3) compared with the pre-existing situation (i.e., the PIC73 network). Furthermore, the PIC73_Vd network was the only one showing *Chloroflexi* (genus *Ktedonobacter*) and *Plantomycetes* (genus *Bythopirellula*) as keystone taxa (Table 4). Conversely, the PICF7_Vd network displayed a decrease of modularity and an important increase of total links compared with the situation observed prior to the inoculation with the pathogen (i.e., the PICF7 network, Table 3). Both networks showed a decrease in the distance among modules (GD) and an increase of connectivity (avgCC), more pronounced in the case of the PICF7_Vd treatment. Besides, these two networks presented an increase of PEP, similarly to the scenario observed in the Vd network. Furthermore, they presented two identical keystones (*Proteobacteria/Bradyrhizobiaceae* and *Actinobacteria/Jatrophihabitans*) (Table 4). The topology of these networks shares some similarities to that of the control (e.g., all showed one of the major modules with an overwhelming presence of positive interactions; Fig. 3). The detailed analysis of these modules revealed very similar compositions. They shared 14 bacterial taxa, mostly belonging to the genera *Actinoplanes*, *Chryseolina* and *Terrimonas*. Moreover, representatives of *Actinoplanes* and *Chryseolina* were module hubs in the three networks (Table 4).

Co-occurrence networks of the microbial communities unveiled for each of the treatments considered in this study: control (CON), *Verticillium dahliae*-inoculated (Vd), *Paenibacillus polymyxa*-treated (PIC73), *P. polymyxa/V. dahliae*-inoculated (PIC73_Vd), *Pseudomonas simiae*-treated (PICF7), and *P. simiae/V. dahliae*-inoculated (PICF7_Vd) plants. The modular layout of the networks is shown, with the nodes coloured according to their phylum. The green and red lines (links) indicate positive and negative interactions, respectively. Rhombi and arrowheads represent module hubs and connectors, respectively.

**Table 3:** Main topological properties of co-occurrence networks of the different treatments examined in this study

Treatment	Similarity threshold	Total nodes	Total links	R^2^ of power-law	Average degree (avgK)	Average clustering coefficient (avgCC)	Average path distance (GD)	Modularity (N. of modules)	Percentage of positive edges (PEP)
CON	0.67	178	283	0.898	3.18	**0.058 b**	**4.860 a**	**0.672 (16) a**	54.77%
PIC73	0.67	168	213	0.838	2.536	**0.028 a**	**6.373 e**	**0.720 (16) b**	49,77%
PICF7	0.67	176	219	0.961	2.489	**0.060 c**	**6.540 f**	**0.760 (18) e**	55.71%
Vd	0.69	140	189	0.926	2.7	**0.093 e**	**6.094 d**	**0.739 (16) c**	62.96%
PIC73_Vd	0.68	168	240	0.893	2.857	**0.077 d**	**5.317 c**	**0.751 (16) d**	62.08%
PICF7_Vd	0.67	183	277	0.87	3.027	**0.109 f**	**5.247 b**	**0.677 (18) a**	62.82%

**Significant differences** (***p*-values < 0.05) among treatments for avgCC, GD and modularity are shown in boldface. The number of modules of each network is indicated between brackets. Treatments: control (CON),* Paenibacillus polymyxa* PIC73-treated (PIC73),* Pseudomonas simiae* PICF7-treated (PICF7),* Verticillium dahliae*-inoculated (Vd),* P. polymyxa***/***V. dahliae*-inoculated (PIC73_Vd) and* P. simiae***/***V. dahliae*-inoculated (PICF7_Vd) plants. The different letters, where present, represent the results of the Tukey ***post-hoc*** test** (*** p*-values < 0.05)**

**Table 4:** Keystone (Module hubs and Connectors) observed in the microbial co-occurrence networks constructed for each treatment

Keystone	Treatment	ASV	Taxonomy	N. module
**Module hubs**	CON	b_ASV00022	*Bacteroidetes/Chryseolinea*	3
		b_ASV00065	*Bacteroidetes/Chryseolinea*	1
		b_ASV00509	*Actinobacteria/Nocardioides*	0
		b_ASV00550	*Bacteroidetes/Niabella*	0
		b_ASV00635	*Verrucomicrobia/Alterococcus*	5
	Vd	b_ASV00271	*Actinobacteria/Micromonosporaceae*	2
		b_ASV00570	*Proteobacteria/Gammaproteobacteria*	3
	PIC73	b_ASV00077	*Proteobacteria/Acidibacter*	2
		b_ASV00092	*Bacteroidetes/Terrimonas*	5
		b_ASV00424	*Bacteroidetes/Fulvivirgaceae*	0
	PICF7	b_ASV00018	*Actinobacteria/Actinoplanes*	0
		b_ASV00264	*Proteobacteria/Rhodanobacter*	5
		b_ASV00468	*Proteobacteria/Mesorhizobium*	5
		b_ASV00557	*Proteobacteria/Rhodoplanes*	1
		b_ASV00635	*Verrucomicrobia/Alterococcus*	4
		f_ASV0008	*Ascomycota*	8
	PIC73_Vd	b_ASV00005	*Proteobacteria/Dongia*	3
		b_ASV00018	*Actinobacteria/Actinoplanes*	0
		b_ASV00065	*Bacteroidetes/Chryseolinea*	0
		b_ASV00283	*Chloroflexi/Ktedonobacter*	2
		b_ASV00431	*Proteobacteria/Bradyrhizobiaceae*	6
	PICF7_Vd	b_ASV00018	*Actinobacteria/Actinoplanes*	0
		b_ASV00038	*Spirochaetes/Spirochaeta*	4
		b_ASV00047	*Proteobacteria/Myxococcales*	3
		b_ASV00077	*Proteobacteria/Acidibacter*	0
		b_ASV00242	*Actinobacteria/Jatrophihabitans*	1
		b_ASV00513	*Verrucomicrobia/Lacunisphaera*	1
**Connectors**	CON	b_ASV00568	*Proteobacteria/Pedomicrobium*	0
		b_ASV00704	*Actinobacteria/Acidimicrobiales*	4
	Vd	b_ASV00005	*Proteobacteria/Dongia*	9
		b_ASV00237	*Proteobacteria/Dyella*	3
		b_ASV00464	*Proteobacteria/Methylocystis*	6
	PIC73	b_ASV00271	*Actinobacteria/Micromonosporaceae*	4
		b_ASV00337	*Verrucomicrobia/Subdivision3*	3
		b_ASV00339	*Proteobacteria/Burkholderiales*	0
		f_ASV0048	*Basidiomycota/Clitopilus*	0
	PIC73_Vd	b_ASV00242	*Actinobacteria/Jatrophihabitans*	1
		b_ASV00611	*Planctomycetes/Bythopirellula*	6
	PICF7_Vd	b_ASV00535	*Proteobacteria/Bradyrhizobiaceae*	6

**ASV, amplicon sequence variants; Taxonomy, phylum/lowest taxonomic level that could be reached for the identification of the keystone; Module number, number of the module in which the keystone was located. Treatments: control (CON),* Paenibacillus polymyxa* PIC73-treated (PIC73),* Pseudomonas simiae* PICF7-treated (PICF7),* Verticillium dahliae*-inoculated (Vd),* P. polymyxa***/***V. dahliae*-inoculated (PIC73_Vd) and* P. simiae***/***V. dahliae*-inoculated (PICF7_Vd) plants**

## Discussion

The plant and its associated microbiome can be regarded as a meta-organism, the so-called “holobiont” [24]. Millions of microbes live in close association with plants, forming a complex community that influences plant growth and health through its collective metabolic activities and host interactions [31]. In this context, the impact of applying BCAs should be evaluated considering not only their separated effects on the pathogen and/or the host but rather on the whole system, which includes the resident microbiota. This knowledge will be instrumental for environmental risk assessment of BCA formulations [24, 32]. The first relevant result of our study was that neither the inoculation with the BCAs nor the subsequent inoculation with *V. dahliae* significantly modified the structure of the microbial communities, as revealed by the analysis of the alpha and beta diversities. This outcome is in accordance with our previous findings on root microbial communities of ‘Picual’ inoculated with *V. dahliae* [34] or banana bacterized with PICF7 [32]. Indeed, in both scenarios (i.e., introduction of a soil-borne pathogen or a beneficial rhizobacteria) no significant changes in the structure of the belowground microbiota were observed.

Concerning the ‘Picual’ root microbiota composition significant changes were detected mostly in taxa with minor relative abundance, except for few interesting differences found in major taxa deserving discussion. At phylum level, a decrease in the relative abundance of *Verrucomicrobia* and *Acidobacteria* was observed after inoculation with PICF7. Previous studies have shown that the abundance of these taxa changes with the availability of labile carbon originated from rhizodeposits, exudates and mucigel [46, 47]. Our results might suggest that introduction of PICF7 modifies the production of plant exudates, thereby affecting the abundance of components of these phyla in ‘Picual’ roots. Recently, the ability of an inoculant to modify the rate and composition of root exudates as an indirect biocontrol mechanism has been described [48]. Another significant change in our study was the decrease of *Glomeromycota* in plants inoculated only with *V. dahliae*, confirming our previous results [34]. This may suggest a strong competition for space and nutrients between pathogenic microorganisms and arbuscular mycorrhizal fungi (*Glomeromycota*) in roots, as reported in peanut (*Arachis hypogaea* L.) [49], pea (*Pisum sativum* L.) [50] and olive [51]. At the genus level, a reduction in the relative abundance of *Dyella* was observed in plants inoculated only with the pathogen, in accordance with studies reporting a decrease in the relative abundance of this bacterium in rice infected with *Burkholderia glumae* [52] and in wheat inoculated with *Rhizoctonia solani* [53]. It is tempting to speculate that *Dyella* may play a role as indicator of stress for the olive root microbial community upon *V. dahliae* inoculation. At the first sampling time [15 DAB], and regardless of whether or not the pathogen was present, plants inoculated with PICF7 showed an increase of *Streptacidiphilus*, a genus of acidophilic *Actinobacteria* that are well-known for their antifungal activity [54]. A recent study has reported the antibacterial and antifungal ability of eleven species of this genus through a comparative genome analysis [55]. At the same sampling time, plants inoculated with PICF7 and subsequently inoculated with *V. dahliae* showed a significant increase of *Novosphingobium*. Some species of this genus are known for their metabolic versatility and involvement in cell-cell signalling [56]. Indeed, several species of this genus are able to produce chemical signals to their surroundings thereby activating population-wide responses leading to the coordination of gene activation or repression in response to environmental cues [56, 57]. Plants of this treatment also showed a decrease of *Rhizoctonia* at 15 DAB, compared with plants only bacterized with PICF7, although the relative abundance of this genus gradually increased at later sampling times. This could suggest antagonism between this fungus and *V. dahliae*, as previously reported in antagonism tests [58]. Interestingly, the opposite trend was observed in plants bacterized with PICF7 [i.e., an increase of *Rhizoctonia* at 15 DAB followed by a decrease later on]. Altogether, these results seem to suggest the ability of PICF7 to recruit taxa either aiding to directly antagonize *V. dahliae* [i.e., antifungal activity] or able to activate a response in the holobiont due to an emerging stress (i.e., presence of the pathogen).

Relating to the impacts of strain PIC73, it is worth mentioning the increase in relative abundances of *Rhizobium* and *Flavobacterium* at 15 DAB. Both genera are well-known PGP rhizobacteria [59–61]. Thus, it can be argued that the presence of PIC73 facilitates the recruitment of beneficial rhizobacteria able to stimulate the plant’s growth by mechanisms such as [micro]nutrients mobilization and suppression of pathogens. Indeed, some species of *Flavobacterium* showed biocontrol activity against *Phytophthora capsici* in pepper [61] and *Clavibacter michiganensis* in tomato [62]. It is tempting to speculate that one of the underlying biocontrol mechanisms exerted by these BCAs could be the consequence [side effect] of subtle modifications on the taxonomic profile of the olive root microbiota. In both cases the recruitment of *V. dahliae* antagonists and/or beneficial microorganisms seemed to be crucial, although each of the BCA facilitated/mediated the “enrolment” of different taxa. It is worth mentioning that strain PIC73 displays a broad antagonist activity [7]. The in situ antibiosis exerted by this rhizobacteria may be thus relevant not only because of the direct inhibition of *V. dahliae*, but also due to changes in the resident microbiota to better cope with the pathogen. In contrast, and without excluding the involvement of antibiosis and modifications of the indigenous microbiota, niche competition and induction of host defence responses seem to play a more decisive role in VWO biocontrol exerted by strain PICF7 [20, 21].

The ASVs corresponding to each of the introduced BCA were not consistently found in BCA-treated samples. Nevertheless, ASV00685 (i.e., strain PICF7) was detected in some samples of PIC73-treated and *V. dahliae*-inoculated plants. This outcome is not totally unexpected, since PICF7 is a natural inhabitant of ‘Picual’ roots and was originally isolated form nursery-produced plants of this cultivar [12]. It is plausible to think that both BCAs experienced a rapid decline over time under our experimental conditions, hindering their detection by the sequencing approach conducted. This result agrees with previous studies reporting a transient establishment and subsequent decline of microbial inoculants. For instance, *Pseudomonas jessenii* and *Serratia plymuthica* experienced a sharp decrease of their relative abundances in the lettuce rhizosphere two weeks after the inoculation of these BCAs [63]; two strains of *P. fluorescence* almost disappeared in the rhizosphere of cucumber seven days after bacterization [30]; or PICF7 showed a rapid decrease in its relative abundance two days after being inoculated in banana roots [32]. Furthermore, considering that alpha and beta diversities did not significantly change and that most of the relevant taxonomical changes occurred at 15 DAB, the impact caused by the introduction of the two BCAs on the indigenous microbiota can be regarded as minor and just taking place during a short period of time after bacterization.

The most outstanding impact after BCA treatment and/or *V. dahliae* inoculation was found in the interactions among components of the belowground ‘Picual’ microbiota, unveiled through co-occurrence network analysis. The network of the plants inoculated only with *V. dahliae* showed a severe decrease in complexity [i.e., reduction of total nodes, links and avgK] compared with the control. Our previous findings were also supported by the fact that GD increased, showing that the native microbial population mitigated the pathogen’s deleterious impact by reducing the number of interactions between modules [34]. The analysis of the PIC73 and PICF7 networks revealed that their effects on the ‘Picual’ root microbiota differed notably. On the one hand, bacterization with PIC73 provoked a diminution of PEP. Recent studies related negative and positive connections with competition and cooperation, respectively [64–66]. This result suggests that the presence of PIC73 increased the number of competitive interactions among the olive root microbiota members compared to the control. This modification could enhance the ability of the microbial community to cope with the *V. dahliae* invasion. Indeed, the study of the rhizo-microbiome of eggplants has demonstrated that a network with more negative interactions better resisted to *Ralstonia solanacearum* infection, compared with a network with higher PEP [67]. On the other hand, the network of PICF7-bacterized plants presented a notable increase of modularity and no connectors, compared with the control. Modularity minimizes the effects of local perturbations on the system as a whole by confining perturbations and damage at a local level [65]. Thus, we can argue that PICF7 inoculation increased the compartmentalization [modules] among members of the microbial community as a strategy to maintain its stability, making it able to better cope with the pathogen. Therefore, the hypothesis to-be-tested in this study has been confirmed: the bacterization with PICF7 or PIC73 produces distinctive alterations in the ‘Picual’ root microbial community. Particularly, each of the BCAs showed the ability to recruit different microbial taxa to help the ‘Picual’ belowground microbiota to confront *V. dahliae* [see above]. But more importantly, they generated different topologies of the co-occurrence networks. Interestingly enough, and even though modifications largely differed, changes in the networks [together with minor alterations in the microbiota composition] did not mean that biocontrol was compromised. This suggests that both BCAs assist the olive holobiont to cope with the pathogen altering the root microbiota in characteristic but equally effective ways. However, additional biocontrol mechanisms cannot be ruled out as mentioned above. After challenging PIC73-treated plants with the pathogen, the resulting PIC73_Vd network increased the modularity and shifted the keystones taxonomy. Thus, bacteria belonging to low abundant phyla (i.e., *Chloroflexi*, *Planctomyces*) acted as module hubs and connectors, unlike the situation observed in control and PIC73 networks where taxa of high abundant phyla (i.e., *Proteobacteria, Bacteroidetes, Verrucomicrobia*) were keystones. This suggests that low abundant taxa may play an important role in maintaining the structure and function of the network to face an incoming perturbation (i.e., *V. dahliae*). It has been proposed that dominant and minor species might temporally “switch” their roles to deal with sudden environmental stimuli [68]. Thus, network analyses can provide key information to determine the role that minor representatives within the microbial community may play in response to pathogen invasion. Moreover, in our case, it may offer clues on the biocontrol mechanism involved. Indeed, the suggestion that PIC73 may confront *V. dahliae* mainly through antibiosis (see above) could be supported by the higher abundance of *Chloroflexi* and *Planctomyces*, genera which are well-known to resist a broad spectrum of antibiotics [67–71]. Furthermore, the increase in modularity may be related to a restructuring of the community to minimize the effects of the perturbation caused not only by the inoculation of the pathogen but also by the previous effects caused by PIC73. Conversely, the PICF7_Vd network showed a significant decrease of modularity and increase of complexity, suggestive of a network with many highly connected taxa [nodes and modules] thereby providing more stability [72]. The increase of complexity in this network suggests an increase of interactions/cooperation within the microbial community, probably among the beneficial taxa recruited by this BCA to cope with the presence of the pathogen [see above]. The presence of a *Bradyrhizobiaceae* representative as a connector may support this hypothesis. It is interesting to note that all the networks inoculated with *V. dahliae* presented high PEP, suggesting more cooperation among community members [64, 65]. This could be a likely consequence of the stress caused by the presence of the pathogen. Increase of positive interaction in the rhizosphere microbiome of *Vicia faba* under saline stress has been reported as well [73].

## Conclusion

**Fig. 4:**
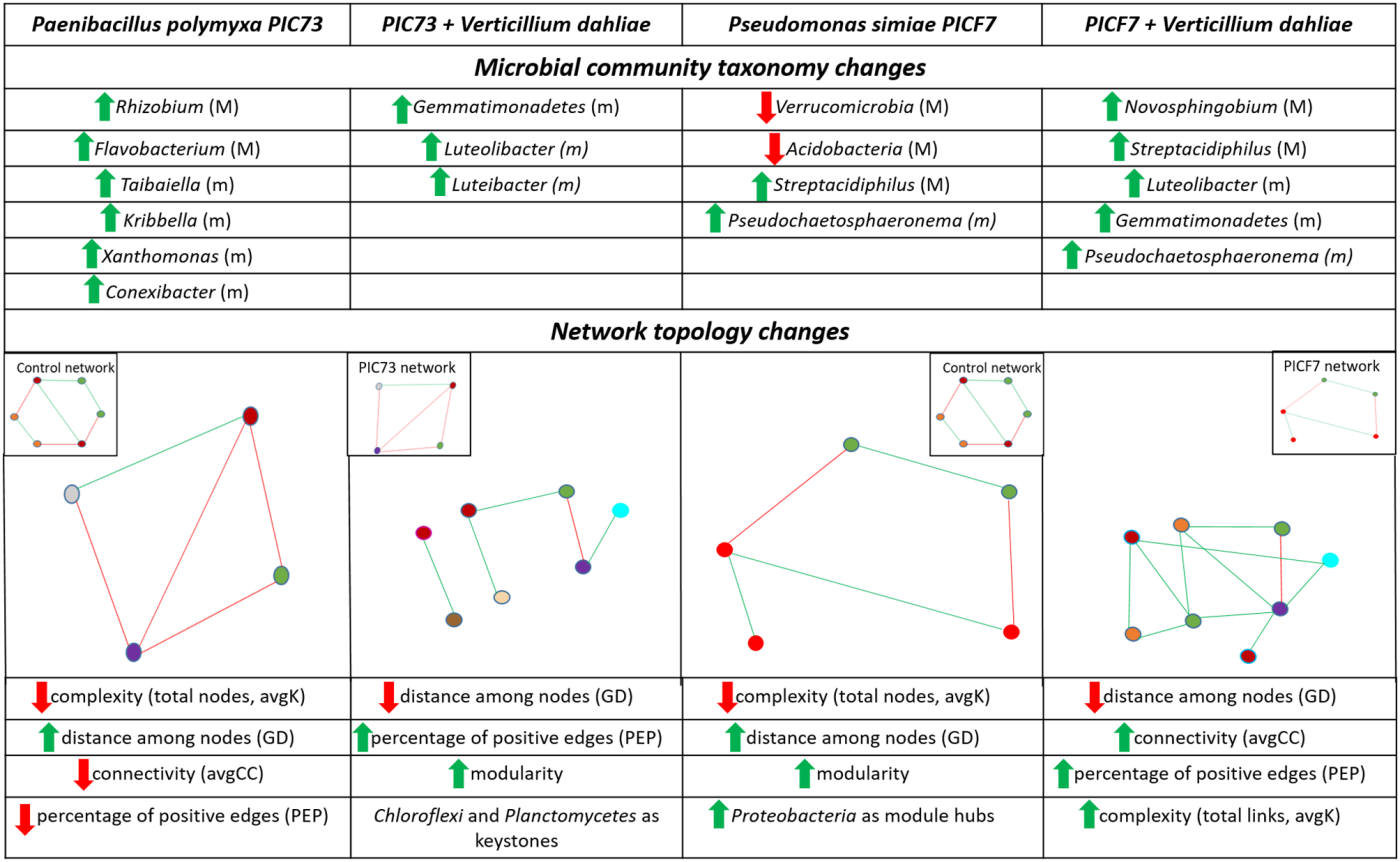
Schematic summary of the major results of this study. (In the upper table, phyla and genera that showed significant changes in relative abundance, at “Treatment” level, after bacterization/inoculation compared to the control treatment are indicated. Changes in major (M) and minor (m) abundant taxa are indicated. Relevant changes in co-occurrence network topologies due to the different treatments are also shown schematically. In the network diagrams, red and green edges represent negative and positive interactions among modules (coloured circles), respectively. The insets within each network scheme refer to the network to which comparisons were made. Different colours of the modules represent differences in keystone taxa. In the bottom table, decrease (red arrows) or increase (green arrows) of the indicated network parameters are shown compared to the network reported in the insets of each scheme)

The two examined BCAs did not decisively alter the ‘Picual’ root microbiota at structural or compositional levels. More precisely, most of the changes observed were limited to minor taxa (Fig. 4) and at early time after bacterization. This outcome may have important practical consequences regarding future applications of these BCAs. For instance, the low/null environmental impact (i.e. minor or transient effects on the root-associated microbial diversity) may facilitate their release in olive orchards as a sustainable approach within a VWO integrated management strategy. Furthermore, these BCAs either alone or in combination with other beneficial rhizobacteria (i.e. as part of synthetic communities) could also be useful in breeding programs for VWO resistance, as well as during the nursery propagation stage to produce plants more effective to confront future attacks of the pathogen under field conditions and from the holobiont perspective. However, while the structure and composition of the olive bewlowground microbiota was not significantly altered, relevant changes in the topology of the co-occurrence networks were found after bacterization. The presence of PIC73 increased the competitive interactions within the ‘Picual’ microbiota, while PICF7 inoculation provoked an increase in compartmentalization of the network [Fig. 4]. These results, together with the few taxonomic changes observed in major taxa, demonstrate the differential impact caused by the BCAs, and may help to better understand their distinct biocontrol strategies against *V. dahliae*. This work confirms the importance of co-occurrence network analysis to detect the actual impacts (beyond potential changes in structure and composition) caused by external factors/perturbations on microbial communities. Moreover, this methodological approach would be instrumental to analyse the eligibility of new BCA inoculants.

### Electronic supplementary material

Below is the link to the electronic supplementary material.

Supplementary Material 1

Supplementary Material 2

Supplementary Material 3

Supplementary Material 4

Supplementary Material 5

Supplementary Material 6

## Data Availability

The datasets generated and analysed during the current study are available in the NCBI Sequence Read Archive (SRA) under the BioProject number PRJNA856429.
