# Supplementary material for: Co-occurrence network analysis unveils the actual differential impact on the olive root microbiota by two Verticillium wilt biocontrol rhizobacteria
Source: Environ Microbiome. 2023 Mar 22;18:21. doi: 10.1186/s40793-023-00480-2 (PMC10035242; doi:10.1186/s40793-023-00480-2)
Supplement: Supplementary file 5 — Supplementary Material 5 [file 40793_2023_480_MOESM5_ESM.pdf]

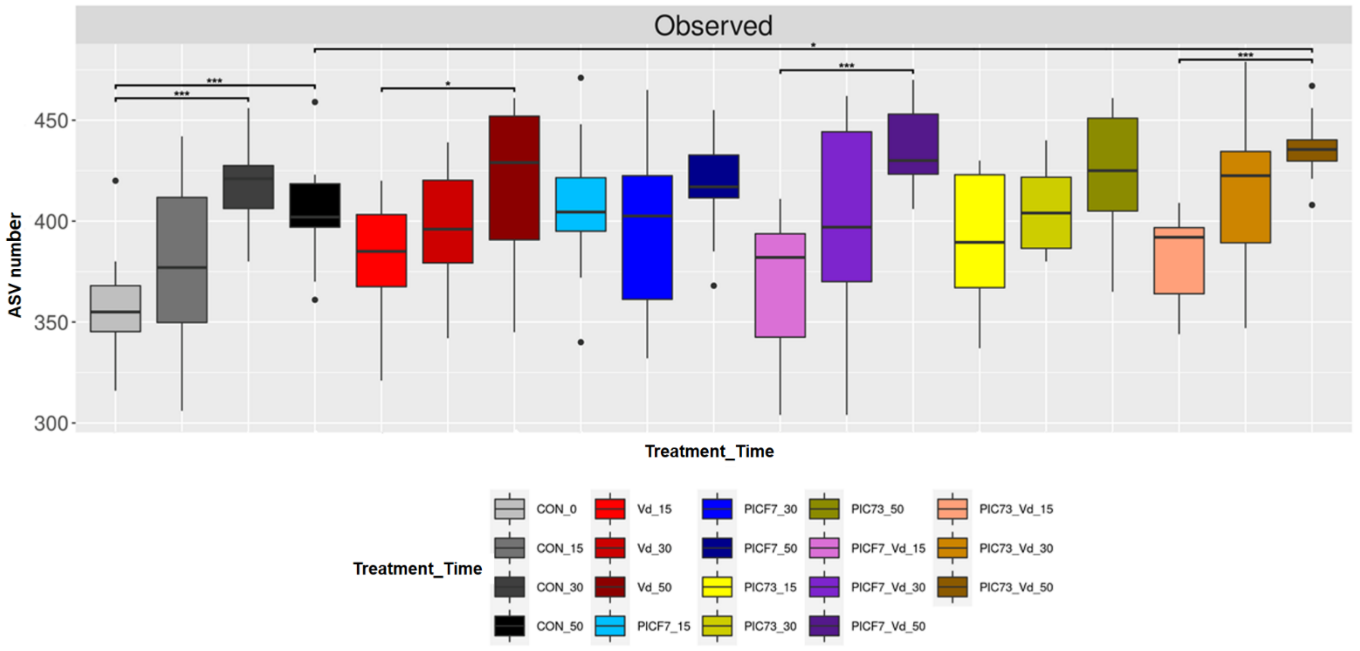

Figure S1. Box plot of the α-diversity index Observed richness for the bacterial community at each sampling time (0, 15, 30, 50 days after bacterization) of control (CON), *V. dahliae*-inoculated (Vd), *Pseudomonas simiae*–treated (PICF7), *P. simiae* PICF7/*V. dahliae*-inoculated (PICF7_Vd), *P. polymyxa*-treated (PIC73) and *P. polymyxa/V. dahliae*-inoculated (PIC73_Vd) plants. The statistical differences resulted by the Dunn post-hoc test are represented by asterisks (level of significance: *p < 0.05; **p < 0.01; ***p < 0.001).

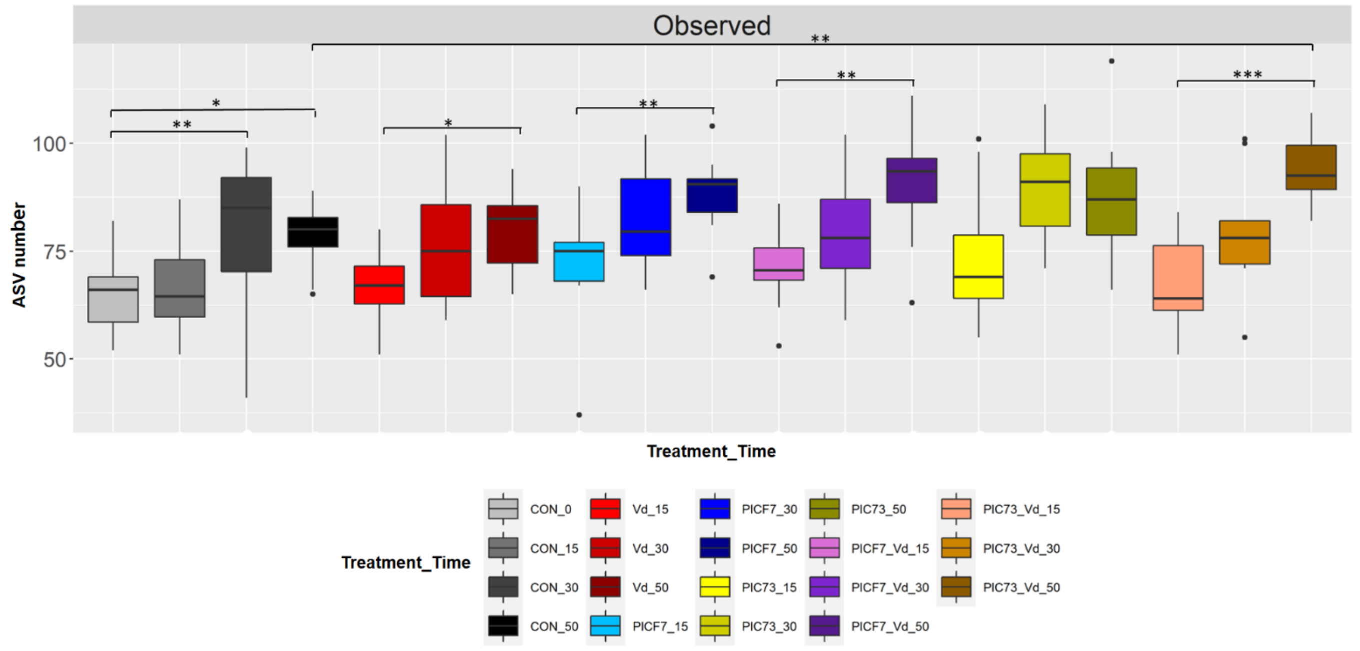

Figure S2. Box plot of the α-diversity indices Observed richness for the fungal community at each sampling time (0, 15, 30, 50 days after bacterization) of control (CON), *V. dahliae*-inoculated (Vd), *Pseudomonas simiae*–treated (PICF7), *P. simiae/ V. dahliae*-inoculated (PICF7_Vd), *P. polymyxa*-treated (PIC73) and *P. polymyxa/ V. dahliae*-inoculated (PIC73_Vd) plants. The statistical differences resulted by the Dunn post-hoc test are represented by asterisks (level of significance: *p < 0.05; **p < 0.01; ***p < 0.001).

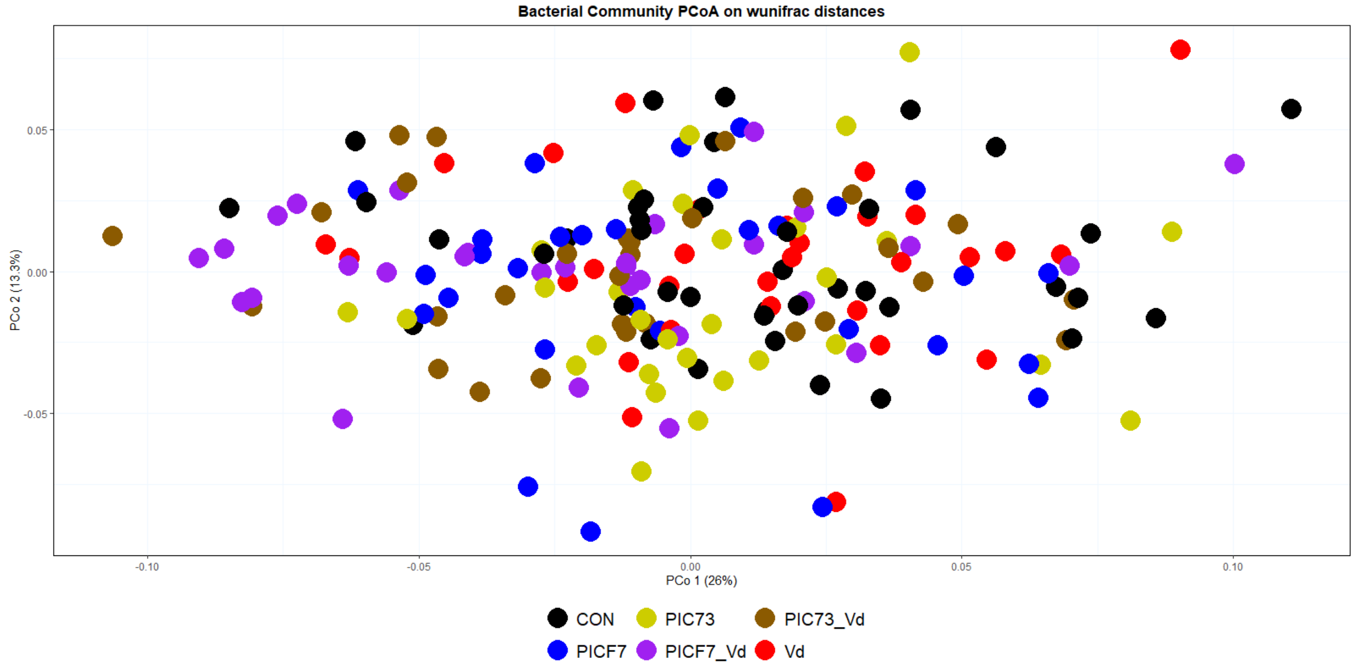

Figure S3. PCoA analysis with the Weighted-Unifrac distance of control (CON), *V. dahliae*-inoculated (Vd), *Pseudomonas simiae*–treated (PICF7), *P. simiae/ V. dahliae*-inoculated (PICF7_Vd), *P. polymyxa*-treated (PIC73) and *P. polymyxa/ V. dahliae*-inoculated (PIC73_Vd) plants.

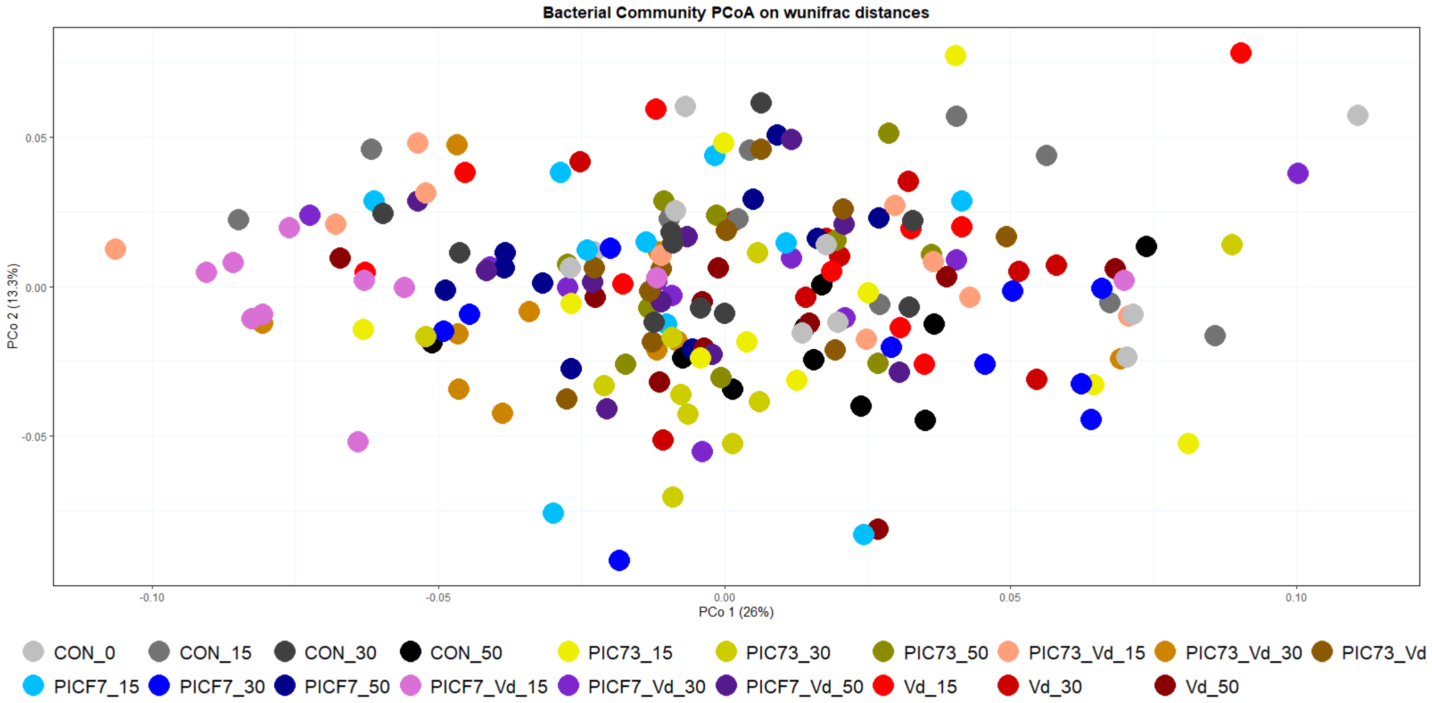

Figure S4. PCoA analysis of the bacterial community with the Weighted-Unifrac distance for the bacterial community at each sampling time (0, 15, 30, 50 days after bacterization) of control (CON), *V. dahliae*-inoculated (Vd), *Pseudomonas simiae*–treated (PICF7), *P. simiae/ V. dahliae*-inoculated (PICF7_Vd), *P. polymyxa*-treated (PIC73) and *P. polymyxa/ V. dahliae*-inoculated (PIC73_Vd) plants.

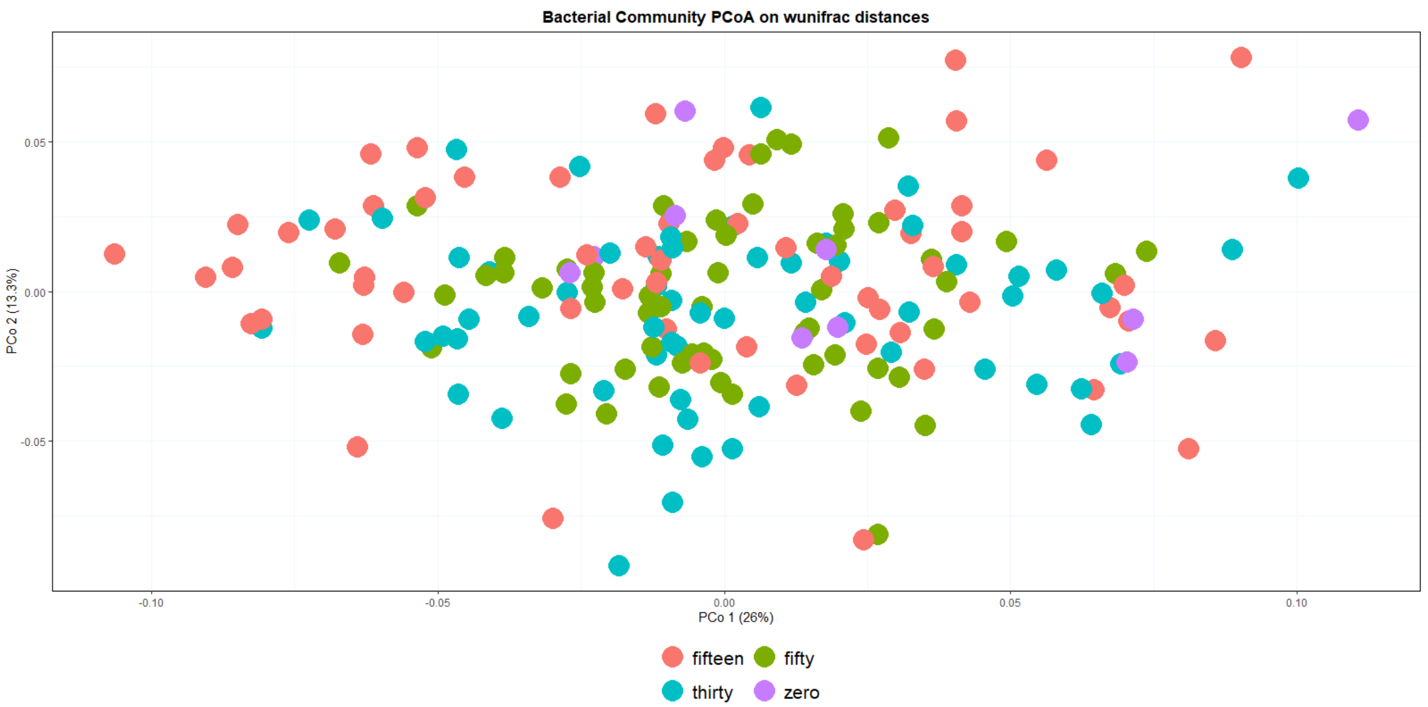

Figure S5. PCoA analysis with the Weighted-Unifrac distance for the factor “time” considering all the sampling times: zero (0), fifteen (15), thirty (30) and fifty (50) days after bacterization.

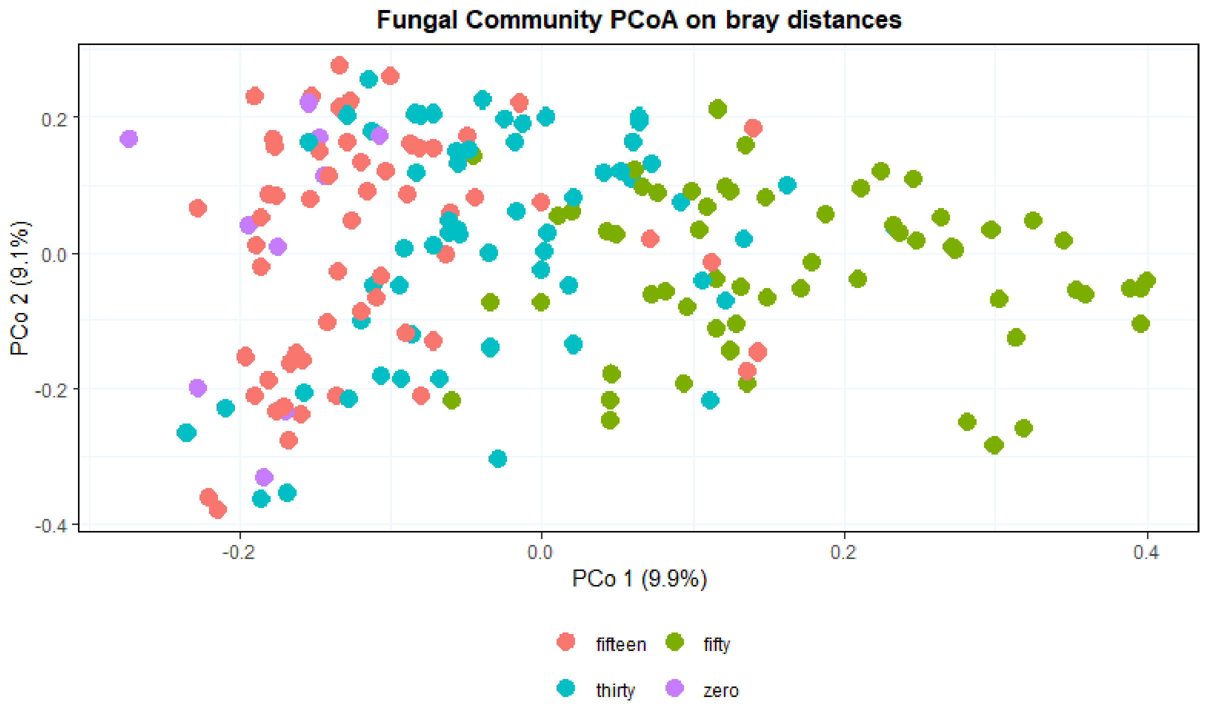

Figure S6. PCoA analysis with Bray Curtis distance for the factor “time” considering all the sampling times: zero (0), fifteen (15), thirty (30) and fifty (50) days after bacterization.

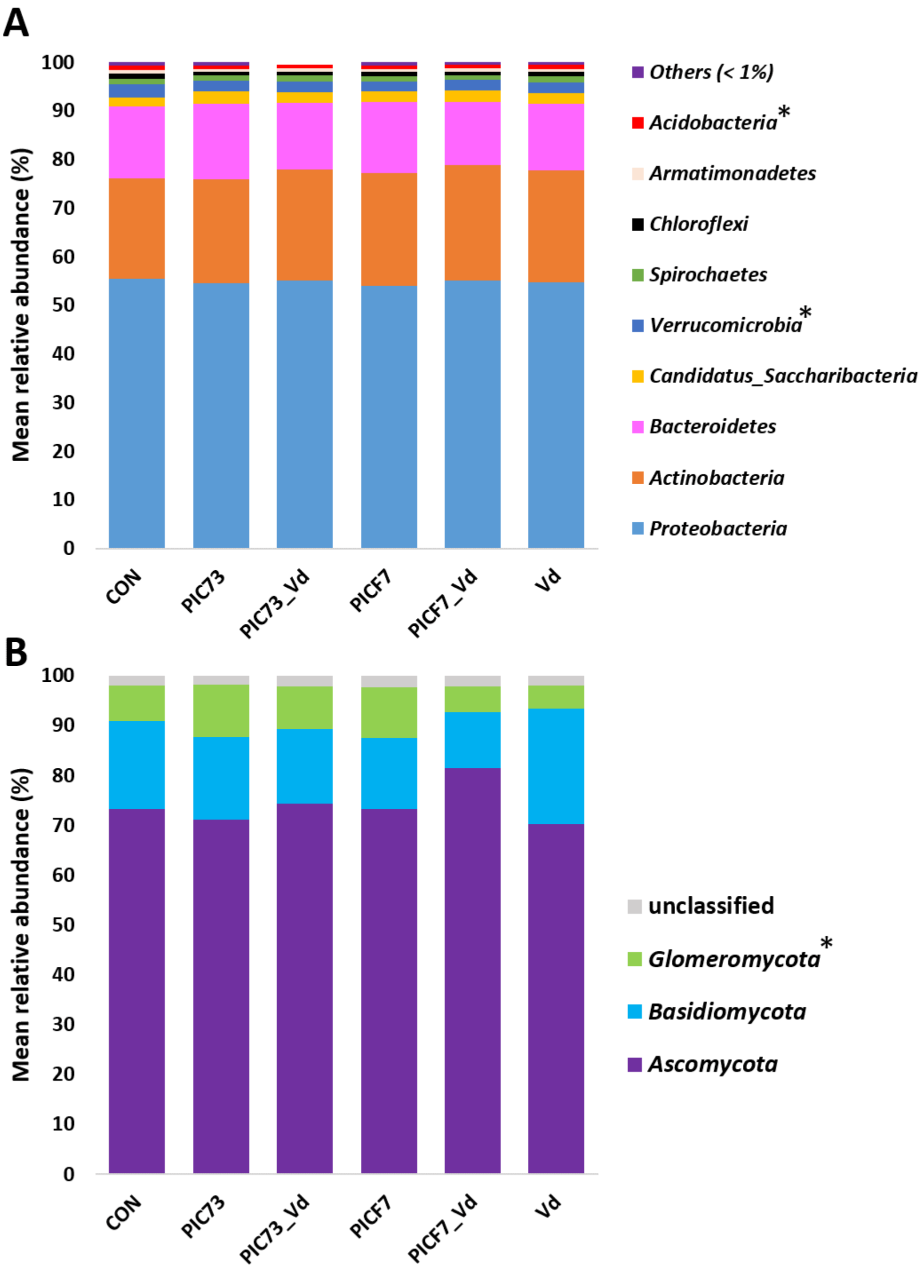

Figure S7. Taxonomy profiles at phylum level of the olive 'Picual' root-associated microbiota upon treatment with two biological control agents and in the absence or presence (subsequent inoculation) of *Verticillium dahliae*. Bacterial (A) and fungal (B) communities at phylum level for the different treatments examined: control (CON), *Paenibacillus polymyxa*-treated (PIC73), *P. polymyxa/V. dahliae*-inoculated (PIC73_Vd), *Pseudomonas simiae*-treated (PICF7), *P. simiae/V. dahliae*-inoculated (PICF7_Vd) and *V. dahliae*-inoculated (Vd) plants. Only the phyla with relative abundance > 1% for bacteria and 0.1% for fungi are shown (n = 10). Asterisks indicate the phyla that showed significant differences (ANCOMB p < 0.05) by the taxonomical analysis (see the main text).

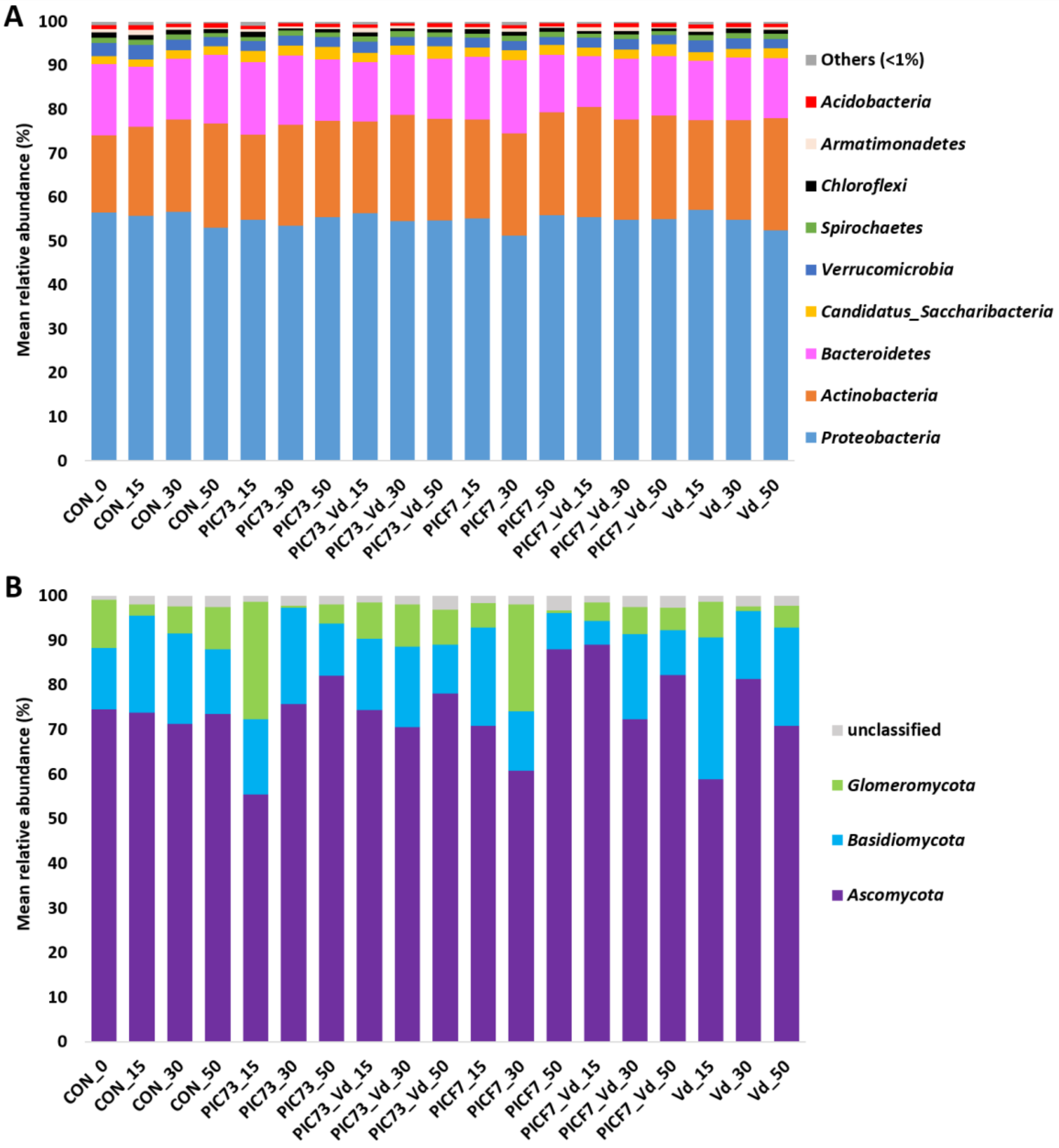

Figure S8. Taxonomy profiles at phylum level of the olive 'Picual' root-associated microbiota upon treatment with two biological control agents and in the absence or presence (subsequent inoculation) of *Verticillium dahliae* at each sampling time (0, 15, 30, 50 days after bacterization). Bacterial (A) and fungal (B) communities at phylum level for the different treatments examined: control (CON), *Paenibacillus polymyxa*-treated (PIC73), *P. polymyxa/V. dahliae*-inoculated (PIC73_Vd), *Pseudomonas simiae*-treated (PICF7), *P. simiae/V. dahliae*-inoculated (PICF7_Vd) and *V. dahliae*-inoculated (Vd) plants. Only the genera with relative abundance > 1% for bacteria and 0.1% for fungi are shown (n = 10).

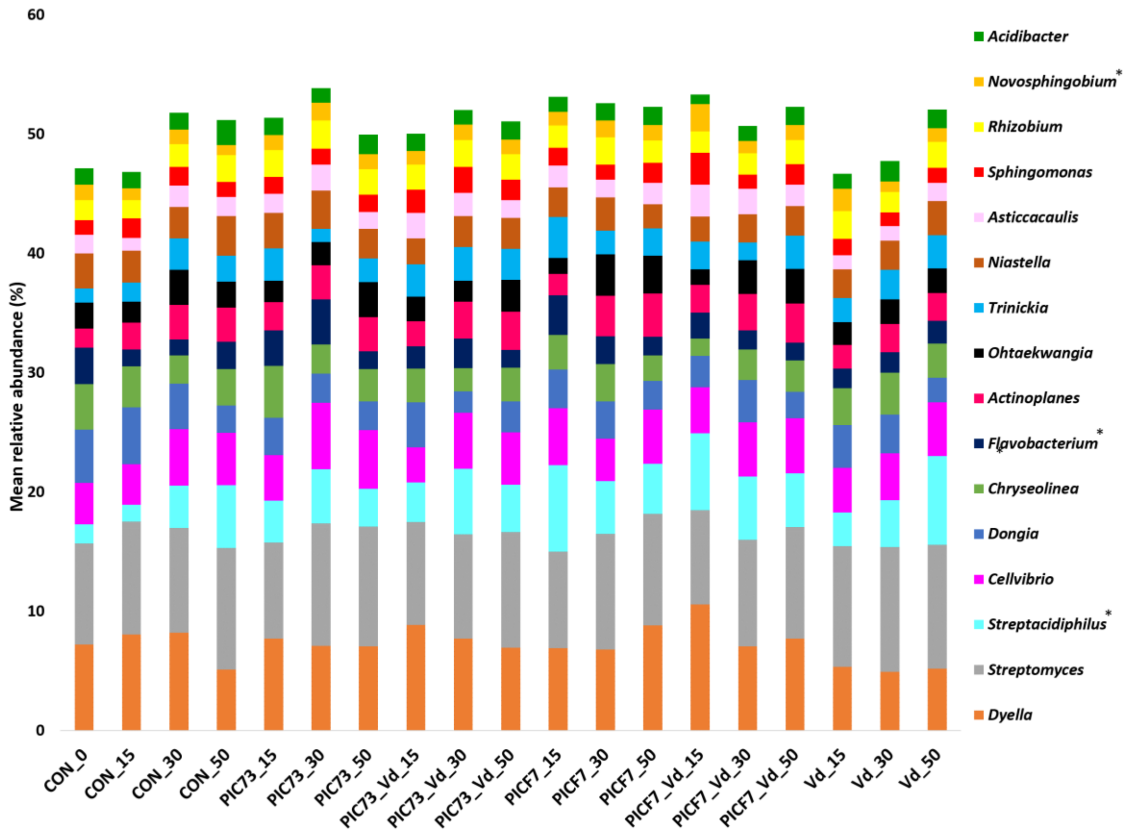

Figure S9. Taxonomic profiles at genus level of the bacterial community at each sampling time (0, 15, 30, 50 days after bacterization) of control (CON), *Paenibacillus polymyxa*-treated (PIC73), *P. polymyxa/V. dahliae*-inoculated (PIC73_Vd), *Pseudomonas simiae*-treated (PICF7), *P. simiae/V. dahliae*-inoculated (PICF7_Vd) and *V. dahliae*-inoculated (Vd) plants. Only the genera with a relative abundance > 2% are shown (n = 10). Asterisks indicate the genera that showed significant differences (ANCOMB $p < 0.05$) by the taxonomical analysis (see the main text).

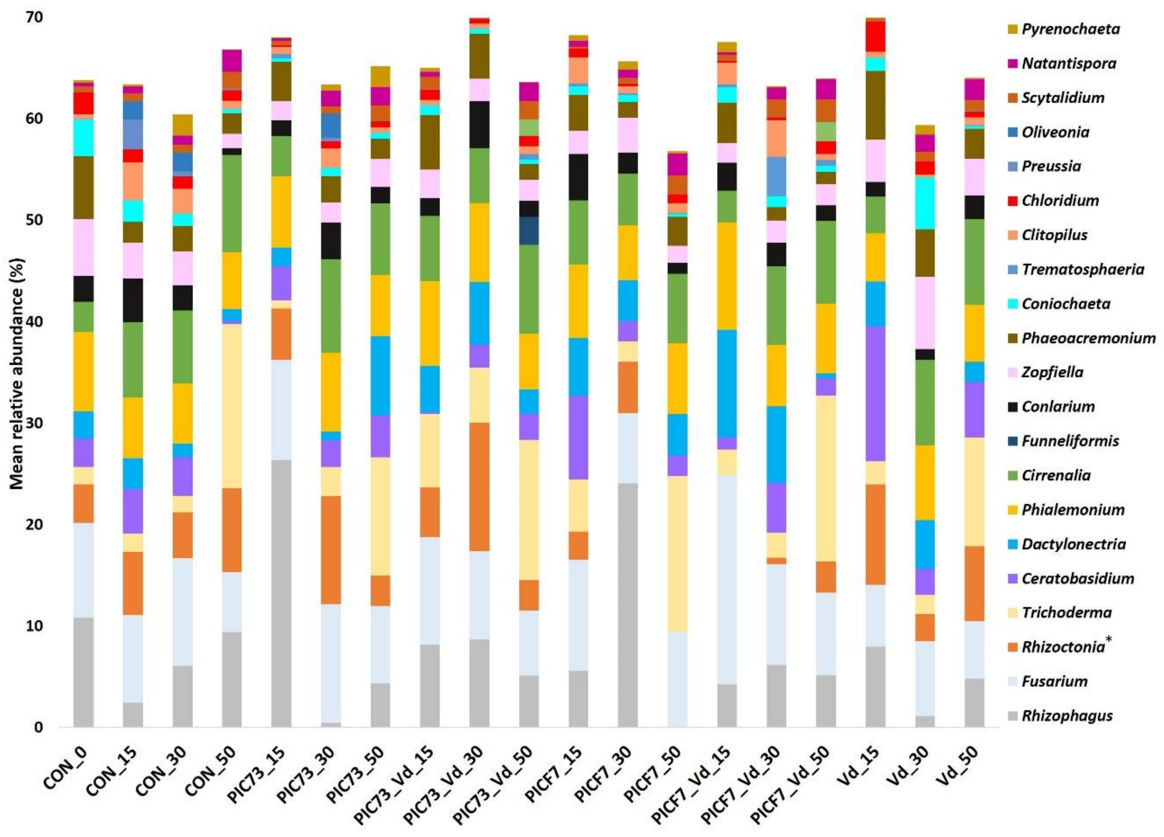

Figure S10. Taxonomic profiles at genus level of the fungal community at each sampling time (0, 15, 30, 50 days after bacterization) of control (CON), *Paenibacillus polymyxa*-treated (PIC73), *P. polymyxa/V. dahliae*-inoculated (PIC73_Vd), *Pseudomonas simiae*-treated (PICF7), *P. simiae/V. dahliae*-inoculated (PICF7_Vd) and *V. dahliae*-inoculated (Vd) plants. Only the genera with a relative abundance > 2% are shown (n = 10). Asterisk indicates the genus that showed significant differences (ANCOMB $p < 0.05$by the taxonomical analysis (see the main text).
